# Using diapause as a platform to understand the biology of dormancy

**DOI:** 10.1098/rsob.250104

**Published:** 2025-08-20

**Authors:** Nathaniel A. Sweet, Chi-Kuo Hu

**Affiliations:** ^1^ Department of Biochemistry and Cell Biology, Stony Brook University, Stony Brook, NY, USA; ^2^ Graduate Program of Molecular and Cellular Pharmacology, Stony Brook University, Stony Brook, NY, USA

**Keywords:** diapause, developmental suspension, metabolic depression, dormancy

## Abstract

Diapause is a fascinating form of biological dormancy that is employed by a broad array of animals as a survival strategy to endure adverse environmental conditions. This unique dormant state can suspend organismal development until a more favourable condition arises, giving the species the greatest chance to survive as a whole. Remarkably, while following the same principle of suspending development, diapause exists in different forms and can occur at various stages before reaching the adult form. Functionally, with multiple evolutionary origins across the animal kingdom, diapause demonstrates the ability to respond to diverse environmental challenges while converging to maintain the same core function of suspending development. At the physiological level, these different diapause states share a similar metabolic adaptation to conserve resources and energy throughout dormancy. Underneath, the same genes have been repeatedly identified as regulators and effectors of diapause at different developmental stages in both invertebrates and vertebrates. This suggests the presence of a conserved molecular programme comprised of the same set of key genes repeatedly reprogrammed and utilized at the core of diapause. The knowledge of diapause from the organismal to molecular levels, together, should serve as a useful window to better understand the biology of dormancy.

**Figure:**
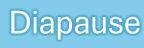

## 1. Introduction

As animals frequently encounter environmental challenges in the wild, many have evolved forms of dormancy to survive through these harsh periods [1]. Diapause is a unique dormant state in which animals can suspend their development until the environment turns favourable [2]. This feature allows animals, as a species, to not only survive the harsh conditions but also time the birth or maturation of their next generation to more favourable conditions.

With its powerful function, diapause is a broadly employed survival strategy in animals. While maintaining the same development-suspending dormant feature, diapause has been observed in various biological contexts, occurring at different developmental stages, responding to different physicochemical cues and addressing different environmental challenges [3]. This remarkable versatility brings up intriguing perspectives to understand diapause across functional, organismal, evolutionary, physiological and molecular levels. The functional impact of diapause also highlights the importance of its crosstalk with organismal developmental and ageing processes. Recent studies have started to fill in the missing pieces of these puzzles, particularly among vertebrates. More importantly, understanding the complex molecular and physiological changes occurring during diapause would not only provide insight into this fascinating adaptation but may also offer insights into other forms of dormancy.

## 2. Diapause is a long-observed phenomenon in nature with a rich scientific history

The discovery of diapause can be traced back to as early as the nineteenth century. The term ‘diapause’ originated from the Greek word *diapausis*, meaning pause. It was first coined by William Wheeler in 1893, for the phenomenon he observed in the eggs of katydids (*Conocephalus brevipennis*) [4]. He observed that these eggs were able to enter a state of developmental arrest during the winter months, where their embryonic development was effectively halted until it began to warm in the spring [4]. Wheeler’s pioneering work has laid the groundwork for recognizing two hallmarks of diapause—as an approach to survive throughout adverse environments, and as a mechanism to time the birth of offspring to a more favourable condition. Soon after Wheeler’s finding, the list of animals that are capable of diapause quickly expanded beyond katydids. In the early twentieth century, the diapause state of developmental arrest was observed and described in a wide variety of invertebrate species [5].

Interestingly, the presence of diapause in vertebrates was actually noticed before Wheeler’s documentation, while under a different name and in a different context. In the early nineteenth century, hunters were aware of an unusual reproductive pattern in the roe deer (*Capreolus capreolus*), which they referred to as ‘silent heat’ [6]. This term described a mismatch between the timing of mating and pregnancy, suggesting an unseen (silent) period of mating. This observation was then recorded by Louis Ziegler in 1843, who documented these reproductive ‘anomalies’ of the roe deer [6]. Approximately 60 years after this initial observation, as the study of diapause in invertebrates advanced, scientists began to draw parallels between the ‘silent heat’ in roe deer and diapause. Franz Keibel first documented the lengthened blastocyst stage of roe deer in 1902 [7]. However, in the next 30 years following his discovery, only two other mammals were confirmed to exhibit a state of diapause, although at the time it was hypothesized that many more mammals may possess the adaptation [8]. This is in drastic contrast to Wheeler’s finding of diapause in katydids which quickly expanded to many other insects within several years [9]. The discrepancy between invertebrate and vertebrate diapause is mostly due to the inherent challenges in observing vertebrate diapause in mammals, as almost all mammals carry their embryos within female bodies. Nevertheless, with time, as of today, diapause has been confirmed to occur in an array of vertebrates, from teleost, to amphibians, reptiles and over 130 mammalian species [3,10–12].

The broad distribution of diapause highlights its significance as an adaptive survival strategy across both invertebrates and vertebrates. These animals—from nematodes to insects, fish, amphibians, reptiles and mammals—have distinct developmental processes and live in diverse habitats. Diapause is thus a dormant state capable of communicating with different developmental programmes, responding to different environmental cues and fitting into different needs for the survival of the species.

## 3. Diapause shows high plasticity of integration into various developmental processes

**Figure 1.**
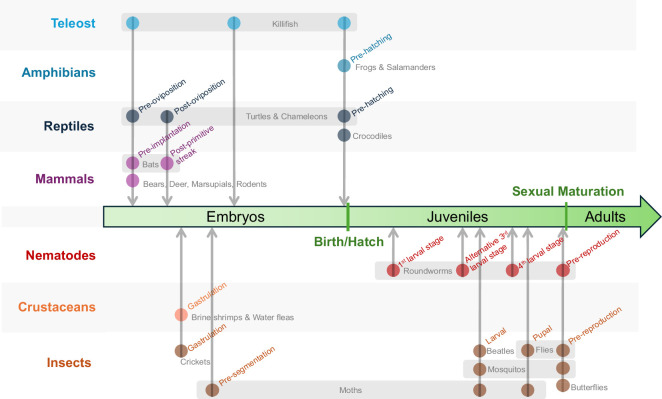
Diapause can occur at similar developmental stages across different species, and multiple times within the same species. Representative organisms are selected. Each dot indicates at least one species within that order where diapause is known to occur. The specific developmental stages of each species are labelled and then vertically aligned to a general developmental timeline between vertebrates and invertebrates. Multiple diapause occurring within the same species, or species from within the same family, are horizontally grouped and labelled. References: teleost [10,18,19], amphibians [20–23], reptiles [24–27], mammals [13,28–34], nematodes [35–38], crustaceans [39–42] and insects [43–52].

One of the most remarkable features of diapause is its ability to integrate into distinct developmental processes across animals. As a later-evolved adaptation, diapause must engage, interrupt, suspend and later resume the preexisting developmental programmes that were already fine-tuned by evolution. Notably, across different organisms, diapause has been observed to occur at various developmental stages both pre- and post-birth, from pre-gastrulation [13–15] (which comprises relatively homogeneous stem cells) to pre-birth [10,16,17] (which comprises differentiated cells and organs) (figure 1). In many cases, diapause also occurs in juveniles before sexual maturation [35,53,54], when organisms have already begun actively living (figure 1). This highlights the plasticity of diapause in interacting with vastly different genetic regulatory networks, cell types, tissues and systems in diverse developmental contexts, as well as its remarkable ability to pause a wide range of developmental processes during the dormant state.

Diapause can occur at different developmental stages within the same species (figure 1, indicated by the grayed horizontal boxes). Many animals have evolved multiple diapause states throughout their life cycle. This helps create varied developmental trajectories to overcome repetitive or different environmental challenges at different times in life. The well-studied invertebrate research organism *Caenorhabditis elegans* (*C. elegans*) presents a good example of this adaptability. To respond to various unfavourable environmental cues, *C. elegans* has four potential diapause entering points—three across the different larval periods (from early to late, L1, L2, L3 and L4) and one before reproduction [35,53–58]. The first diapause occurs within L1 when starved, known as L1 arrest [53]. The second diapause is the well-known dauer, triggered by high population, low food or high temperatures [35]. Dauer serves as an alternative L3 following L1 and L2. Thus, *C. elegans* larvae entering dauer are not developing through L3, providing a different developmental route to L4. The third diapause occurs in early L4 when food is scarce, known as L4 arrest [54]. However, for the L4 larvae already committed to adulthood (as well as the starved adults), they will morph into the adult reproductive diapause, the fourth diapause state of *C. elegans*, with constricted gonad and arrested germline stem cell proliferation [55]. Importantly, after refeeding, *C. elegans* will resume their growth and reproduction with a normal lifespan, indicating the essential protection of diapause and how organisms use multiple diapause stages to hedge the risk from a harsh environment throughout their lives [35,59]. Similarly, many vertebrates also evolved multiple diapause states during their embryonic development. Prime examples are the killifish, which can potentially enter diapause at three developmental stages: pre-gastrulation (diapause I), pre-pharyngula (diapause II) and pre-hatching (diapause III), located at the beginning, middle and end of embryogenesis, respectively [10,18,19,60,61]. Many killifish species live in ephemeral ponds with water present only during the rainy season [10,62,63]. To survive the lengthy drought following the desiccation of the ponds, killifish rely on diapause to suspend their development to live in the form of embryos protected in the drought-resistant eggs until the next rainy season refills their dried-up ponds [10]. The multiple diapause entering points respond to different environmental cues, helping killifish cope with the unpredictable climate of their harsh habitat. As a whole, multiple diapause states also synchronize the birth of the killifish at the beginning of the rainy season and thus maximize the chance of propagation in the ephemeral ponds. This unique yearly cycling survival strategy built on diapause, called ‘annualism’, is also observed in other vertebrates such as amphibians (e.g. *Pseudophryne bibronii* and *Ambystoma opacum*) and reptiles (e.g. *Kinosternon baurii* and *Furcifer labordi*) [20–27,64,65]. While it remains to be learned whether these amphibians and reptiles also employ multiple diapause stages in their annualism, studies have shown that reptiles can initiate diapause at three potential stages: early pre-oviposition, post-oviposition and pre-hatching.

Diapause can also occur at the same developmental stage across different species (figure 1, indicated by the vertical arrows). While diapause can integrate into a broad spectrum of developmental processes, it seems to evolve more frequently at some specific stages, even between species that are evolutionarily remote. As described earlier, *C. elegans* can enter adult reproductive diapause right before reproductive maturation [55]. This pre-sexual maturation diapause can also be observed in a wide range of insects, including fruit flies (*Drosophila melanogaster*), mosquitoes (*Culex pipiens*) and butterflies (*Danaus plexippus*) [43–45]. As reproduction is a resource-demanding process, pausing it allows fruit flies and mosquitoes to conserve energy, extend their life cycle through winter, and only reproduce when environmental conditions are ideal. Monarch butterflies, which migrate 3000 miles annually between north and south, use the same diapause approach to extend their lifespan over the winter migration, so they can get back to the warmer south within one generation to breed (the same route takes three generations in the spring, as monarch butterflies breed along the migrating route) [66,67]. Similarly, among vertebrates, despite having distinct developmental schemes, diapause frequently occurs at the pre-gastrulation stage across diverse species. Most of the known diapause states in mammals (including bears, rodents and marsupials) occur right before embryonic implantation into the uterus [68]. Beyond mammals, while reptiles and killifish are developed in eggs, they can also enter diapause at the pre-gastrulation stage (pre-oviposition in reptiles and post-epiboly stages in killifish) [17,24]. Some reptiles and killifish obtain another frequently shared diapause stage, which occurs right before hatching from the eggs. This diapause functions as the last guardrail, ensuring that hatching is thus not automatic and will proceed only when the fully developed embryos verify the environment is suitable for their birth [10,25].

Across both invertebrates and vertebrates, diapause demonstrates a remarkable ability to interrupt and suspend developmental processes at various stages. This plasticity allows diapause to occur at multiple developmental stages within the same species, yet it also tends to occur more frequently at specific stages across different species. Understanding the underlying mechanisms that drive this plasticity and the preference to engage in specific developmental programmes will be an important area for future research.

## 4. Diapause can be initiated in an obligatory or facultative fashion

Diapause is a physiological response to environmental stressors that threaten the survival of an animal species. The initiation of diapause is thus tightly linked to environmental stress, which can either be regular, such as seasonal changes, or unpredictable, such as food shortages [3]. To accommodate diverse needs, animals can enter diapause obligatorily or facultatively [3,68,69].

Obligatory diapause is initiated regardless of the surrounding environment. It functions as an integral part of the life cycle responding to regular and predictable environmental challenges [3,5]. As a pre-determined decision, obligatory diapause allows the organisms to prepare before the arrival of unfavourable conditions [3,70]. The embryos of some strains of the silkworm moth (*Bombyx mori*) obligatorily enter embryonic diapause for the winter [46,71–73]. This obligatory diapause helps silkworms not only avoid cold temperatures during winter but also synchronize their life cycle with the availability of mulberry trees in the spring, maximizing their chances of survival and growth [72,73]. Similarly, the key diapause in the killifish to survive the drought also seems to be obligatory, as the killifish embryos still enter diapause in the presence of water [10]. In recent years, several killifish species have been established as research organisms in the laboratory, including the African turquoise killifish (*Nothobranchius furzeri*) [74–76] and the South American ocellaris killifish (*Austrofundulus limnaeus*) [77–79]. Their diapause entering pattern remains in a laboratory setup, with consistent temperature and standardized pH neutral buffer in an incubator all year round, completely dissociated from their natural habitats [10].

Facultative diapause, on the other hand, is induced by environmental cues associated with unfavourable conditions when or even before animals encounter them [3,5,9]. It provides animals with flexibility, allowing them to only enter diapause when needed. A well-known example is the dauer stage of *C. elegans* [35,58,80,81]. The decision to enter dauer is made when *C. elegans* senses high population density, low food availability or high temperatures [35,82]. Among monarch butterflies, while migrating the same route, only the population migrating south in winter will initiate reproductive diapause to suspend the development of sexual organs and stay in the juvenile form to survive the long distance, while the populations migrating north in the spring and summer will not [43,67,83]. Using diapause as an overwintering approach is broadly observed across a diverse range of animals, as the changing seasons present one of the most anticipated challenges animals regularly face in the wild [68,84]. Animals frequently rely on photoperiod and temperature as their cues to sense the impending winter and initiate diapause accordingly [85–88]. For instance, the European corn borer (*Ostrinia nubilalis*) enters diapause as larvae when exposed to less than 14 h of daylight, while the Colorado potato beetle (*Leptinotarsa decemlineata*) enters reproductive diapause when temperatures drop below 15°C, regardless of changes in daylight [47,48,89,90]. The embryonic diapause of the Asian tiger mosquito (*Aedes albopictus*), for example, is influenced by both temperature and photoperiod, with both low temperatures and decreased daylight leading mothers to produce eggs undergoing diapause [49,91–93]. The dual requirements may serve as a safeguard against unpredictable weather patterns, such as warmer winters, which could lead to the premature termination of the pupal diapause in species like the blue orchard bee (*Osmia lignaria*), potentially disrupting its synchronization with its food source [94,95]. On the other hand, some rodents and marsupials that have breeding cycles all year round, such as mice (*Mus musculus*) and red kangaroos (*Macropus rufus*), utilize facultative embryonic diapause to properly pace the interval between the births of offspring [68,96,97]. When suckling newborns are present, newly fertilized embryos, which are not implanted into the uterus, will enter diapause before gastrulation. The embryonic development of the incoming offspring is thus suspended until the newborns are grown enough to no longer require nipple sucking, ensuring that all the newborns will have access to milk for growth [28,98–103]. Similarly, some semiannual killifish such as the blue lyretail (*Fundulopanchax gardneri*) can enter diapause pre-pharyngula depending on the population density [104], reflecting a delicate way of utilizing diapause to adapt variable environment for maximizing the chance of survival as a species.

It is important to note that the line between obligatory and facultative diapause might not always be clear. Animals might evolve mechanisms to ‘opt out’ of obligatory diapause. This can be seen in the very first clutch of embryos of the female South American ocellaris killifish (*A. limnaeus*) [105] and the African turquoise killifish (*N. furzeri*) [106]. Unlike latter clutches of embryos, the first clutch shows a higher tendency to skip diapause and develop continuously [105,106]. It is believed to be beneficial to the killifish as these early offspring at the beginning of the rainy season should have time to mature and produce more embryos before the drought, although no ecological studies have occurred to test this hypothesis to date [105]. At the same time, it is also very possible that some diapause states currently considered obligatory might actually be facultative with the cue not yet identified. One good example is that the long-believed obligatory pupal diapause of the cecropia moth (*Hyalophora cecropia*) was later unmasked to be regulated by changes in photoperiod (facultative) [72,107].

## 5. Diapause might be independently evolved multiple times across different animals

Both diapause’s broad distribution across animals and its high plasticity at developmental stages have made the origin of different diapause states a subject of debate for over a century [5]. While it is generally accepted that distantly related species evolved this trait independently, it is less clear whether this holds true for closely related species [108]. This uncertainty is partly because much of our understanding came from comparative biology, where researchers were looking at phylogenic trees to determine the evolutionary relationship of diapause [109,110]. Not every species within the same genus or family exhibits a diapause state, indicating that diapause was either independently evolved with multiple origins or was individually lost from a single origin across different lineages. For example, in mammals, stoats (*Mustela erminea*) and ferrets (*Mustela putorius*) are closely related species that share a common ancestor. However, only stoats exhibit diapause, and both theories have been proposed as potential explanations for the discrepancy [111,112]. Furthermore, the complex distribution of dauer among nematodes, coupled with the absence of known genetic information (excluding *C. elegans*), has led to a debate regarding its evolutionary origin [36,113]. More in-depth analysis, including genetic and molecular profiling, has started to identify the missing links, although there remains a lack of consensus.

Some evidence suggests that certain closely related species may have evolved diapause from a common ancestor [111,114]. Research on mammalian diapause has provided some support for this concept. One study took blastocysts from sheep (*Ovis aries*)—a species that does not naturally exhibit diapause—and placed them into the uteri of mice under diapause conditions. The sheep blastocysts were shown to have some physiological diapause-like changes, such as decreased cellular proliferation and a diapause-like gene expression pattern [114]. The apparent conservation of diapause machinery in naturally non-diapausing sheep embryos may suggest that diapause originally came from a common ancestor before being lost across different lineages [114,115].

Most studies suggest that diapause is the product of convergent evolution, even among closely related species. For example, not all species of *Drosophila* undergo diapause, which seems to be independently evolved within the genus [116,117]. Killifish, as the only teleost known to undergo diapause, serves as another valuable research model for the evolution of diapause. Both the African and South American continents have many killifish genera that include species both with and without diapause [118]. There thus exists an interest in determining the evolutionary history of diapause at different phylogenic levels of killifish. Earlier studies preferred a single origin of killifish diapause before the division of Africa and South America 138 million years ago [107]. However, recent studies have leaned towards the idea of multiple convergent evolutionary events as independent origins of killifish diapause not only between two continents but also among and within different genera in each continent [16,109,119]. Additional data, especially at the molecular level, such as the regulatory signatures of promoters and enhancers across different killifish species, might be needed to reach a conclusion. One of the most striking examples of the independent evolution of diapause is found in the Asian tiger mosquito (*A. albopictus*). Originally incapable of entering diapause, certain mosquito populations rapidly developed this ability within just 35 years of being introduced to seasonally colder environments [120]. These variations highlight the possibility that diapause evolved independently in response to specific environmental pressures and developmental needs. The convergent evolution of different diapause states also reflects a recurring theme of evolutionary reuse, where similar genetic and developmental processes are repurposed to meet the demands of different species. For instance, Forkhead box (FOXO) genes have been implicated in regulating different diapause states across nematodes, mosquitoes, killifish and mice, suggesting that similar genetic pathways may be utilized across species [3].

Whether these different diapause states stem from a common ancestor, convergent evolution, or even a combination of the two remains a complex discussion [3]. As advancements in high-throughput profiling technologies facilitate our understanding of the molecular underpinnings of diapause, we shall gain more insight into the evolutionary history of diapause.

## 6. Diapause research has utilized a broad range of research organisms to obtain an understanding at the molecular level

Diapause has been studied in various research organisms from invertebrates to vertebrates (table 1). While most of our knowledge of diapause comes from invertebrates, a more thorough understanding among vertebrates is gradually emerging. Advancements in mammalian stem cell culturing and the emergence of new vertebrate research organisms, such as annual killifish, have facilitated further in-depth studies.

**Table 1.** Research organisms historically and commonly used for diapause research. A non-exhaustive list of organisms chronologically listed by the order of the time they were first used for diapause research.

research organism	since	developmental phase and stage		initiation	research focus	references
*vertebrates*						
roe deer (*Capreolus capreolus*)	1840s	embryo	pre-implantation	obligatory	diapause discovered in vertebrates, molecular mechanisms	[6,7,13,14]
house mouse (*Mus musculus*)	1910s	embryo	pre-implantation	obligatory	energetics, genetics, hormonal regulation, molecular mechanisms, stem cells	[8,99,121–124]
Bibron’s toadlet (*Pseudophryne bibronii*)	1920s	embryo	pre-hatching	facultative	environmental influence	[20,21]
straw-coloured fruit bat (*Eidolon helvum*)	1960s	embryo	pre-implantation	obligatory	hormonal regulation	[30,31]
mink (*Mustela vison*)	1970s	embryo	pre-implantation	obligatory	evolutionary history, hormonal regulation	[125–128]
Tammar wallaby (*Macropus eugenii*)	1970s	embryo	pre-implantation	facultative and obligatory	hormonal regulation	[8,15,29]
South American Myer's killifish (*Austrofundulus myersi*)	1970s	embryo	pre-pharyngula pre-hatching	obligatory obligatory	diapause staging	[10]
mud turtle (*Kinosternon baurii*)	1970s	embryo	pre-gastrulation pre-hatching	facultative facultative	environmental influence	[26,27,64]
North American salamander (*Ambystoma opacum*)	1980s	embryo	pre-hatching	facultative	environmental influence	[22,23]
South American ocellaris killifish (*Austrofundulus limnaeus*)	1990s	embryo	pre-pharyngula pre-hatching	obligatory obligatory	energetics, evolutionary history, genetics, lipidomics, molecular mechanisms, proteomics	[77–80,105,118,129–131]
blackfin pearl killifish (*Austrolebias nigripinnis*)	2000s	embryo	pre-pharyngula pre-hatching	obligatory obligatory	energetics, evolutionary history	[19,62]
greater short-nosed fruit bat (*Cynopterus sphinx*)	2000s	embryo	post-primitive streak	obligatory	molecular mechanisms	[33,34]
African turquoise killifish (*Nothobranchius furzeri*)	2000s	embryo	pre-pharyngula pre-hatching	obligatory obligatory	energetics, evolutionary history, genetics, lipidomics, molecular mechanisms, proteomics	[74–76,106,109,119]
Labord's chameleon (*Furcifer labordi*)	2000s	embryo	pre-implantation	obligatory	environmental influence, stress resistance	[25,65]
South American duskyfin pearl killifish (*Austrolebias charrua*)	2000s	embryo	pre-gastrulation pre-hatching	obligatory obligatory	evolutionary history, proteomics	[18,61]
*invertebrates*						
katydid (*Conocephalus brevipennis*)	1890s	embryo	gastrulation	facultative	diapause discovered in invertebrates, environmental influence	[4]
silkworm moth (*Bombyx mori*)	1930s	embryo	pre-segmentation	obligatory	energetics, evolutionary history, hormonal regulation, molecular mechanisms	[46,71–74,132–135]
brine shrimp (*Artemia salina*)	1960s	embryo	gastrulation	obligatory	energetics, stress resistance	[42,136]
water flea (*Daphnia pulex*)	1960s	embryo	gastrulation	facultative	environmental influence, maternal factors	[40,41]
monarch butterfly (*Danaus plexippus*)	1970s	juvenile	pre-reproduction	facultative	epigenetics, hormonal regulation, molecular mechanisms	[43,67]
roundworm (*Caenorhabditis elegans*)	1970s	juvenile	1st larval stage alternative 3rd larval stage 4th larval stage pre-reproduction	facultative facultative facultative facultative	genetics, molecular mechanisms, stress resistance	[18,37,53,55–61,80–82,137–152]
house mosquito (*Culex pipiens*)	1980s	juvenile	pre-reproduction	facultative	energetics, evolutionary history, proteomics	[45,153–155]
fruit fly (*Drosophila melanogaster*)	1980s	juvenile	pre-reproduction	facultative	environmental influence, genetics, hormonal regulation	[44,87,117,156,157]
roundworm (*Pristionchus pacificus*)	1990s	juvenile	alternative 3rd larval stage	facultative	evolutionary history	[36,113]

Historically, invertebrates have been the research organisms of choice for finding the molecular underpinnings of diapause. While the wide variety of insects has provided valuable insight into both the plasticity and the systemic regulation of diapause, *C. elegans* dauer has especially advanced our understanding of the underlying genetic and molecular mechanisms of diapause [5,58,158]. First described by Cassada and Russell in 1975, dauer is a diapause state providing an alternative developmental trajectory for *C. elegans* to overcome environmental challenges including high population, low food and hot temperatures [35]. As a well-established canonical research organism, *C. elegans* is equipped with high accessibility and rich resources that have been instrumental in profiling the dauer state. By examining the molecular changes that occur during dauer formation, researchers have identified key genes to either enhance or suppress dauer, collectively called dauer formation (daf) genes [137]. Many daf genes are known to involve important pathways in cells such as nutrient sensing/management and stress resistance, for example, daf-2 (insulin-like receptor), daf-7 (TGF-β), daf−12 (nuclear hormone receptor), daf-15 (RAPTOR), daf-16 (FOXO) and daf-36 (rieske oxygenase) [82,138–145]. The daf genes provided a valuable foundation for understanding the molecular basis of diapause. Importantly, many of the daf genes were later found to also play critical roles in longevity, evidencing the intertwining signalling pathways between diapause and ageing [143,144,146,147]. In fact, daf-2 was one of the very first genes identified to be able to extend the lifespan of *C. elegans*, a critical milestone that eventually initiated the modern research of ageing [146,148–150]. Remarkably, new diapause pathways, such as cold-inducible diapause, are still recently being discovered [151]. Unlike previously established diapause states in *C. elegans* which occur at set developmental stages, *C. elegans* can enter cold-inducible diapause at 4°C at apparently any developmental stage [151]. These advancements have broadened our understanding of diapause mechanisms and established a crucial foundation for future studies across diverse species.

Research into vertebrate diapause has been relatively challenging when compared to invertebrate counterparts [3]. One of the key reasons has been the lack of feasible research organisms for vertebrate diapause. The canonical research organism in vertebrates: mice carry embryos internally hindering the accessibility for *in vivo* studies, and zebrafish (*Danio rerio*) do not have diapause. Consequently, diapause research in vertebrates has traditionally focused on broader systemic factors like environmental and maternal hormonal regulation in mammals rather than the specific molecular mechanisms underlying diapause in embryos [152]. Furthermore, traditionally, the ovaries are removed in mammals to ensure diapause conditions, limiting comprehensive studies [159]. Recent advancements have shifted the focus towards a more mechanistic view of the process. Breakthroughs in culturing mouse embryonic stem cells (mESCs) have allowed researchers to overcome some of the limitations of *in vivo* sampling, providing better access to understanding the molecular aspects of mammalian diapause [102,160]. Furthermore, stem cell culturing of other mammalian species (like humans) has facilitated whether the underlying mechanisms of diapause can be extended to non-diapause species [161].

Beyond mammals, other vertebrate research organisms have also been established to advance the research of vertebrate diapause, noticeably, killifish. Compared to canonical vertebrate research organisms, killifish have the advantageous feature of high accessibility to transparent diapause embryos outside the female body. Historically, various killifish have been utilized to study diapause, including the South American Myer’s killifish *Austrofundulus myersi* in the 1970s, and later the South American ocellaris killifish *A. limnaeus* since the 1990s [10,162]. More recently, the African turquoise killifish *N. furzeri* has generated significant traction and emerged as a powerful research organism, not only for its ability to enter diapause but also for its extremely short lifespan of 6–8 months [163]. This combination of long diapause and short lifespan makes the African turquoise killifish the only vertebrate that can stay in diapause longer than its lifespan. This unique feature allows researchers to study the impact of diapause on the subsequent life of organisms. Given that the signalling pathways of diapause and ageing are frequently intertwined, the African turquoise killifish allows researchers to study not only the vertebrate diapause itself but also its crosstalk with ageing [146,163,164].

The recent establishment of mESCs and killifish as model systems to study diapause has facilitated its characterization at the molecular level. Key regulatory molecules, such as cMyc, Polycomb groups and various miRNAs, have begun to be identified, although the list remains far from complete [37,106,165–169]. Vertebrates generally have a more complex regulatory network than invertebrates and frequently contain multiple gene paralogues corresponding to the single invertebrate gene homologue. Redundant and compensatory mechanisms are thus expected [170]. Significant gaps of knowledge, especially the functional analyses of these candidates, need to be addressed to obtain a more complete picture of the molecular mechanisms of diapause in vertebrates.

## 7. Diapause programmes receive environmental signals frequently through a complex network of hormones

**Figure 2.**
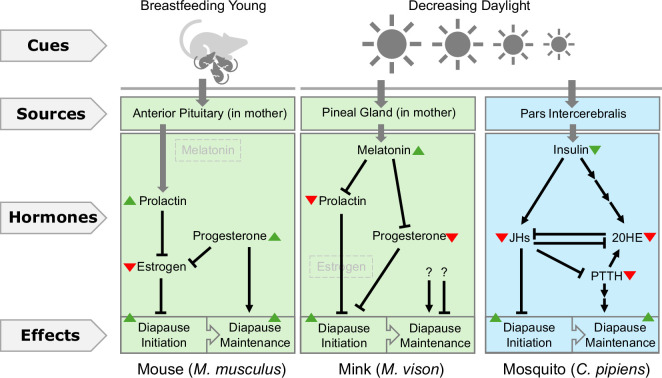
Diapause is frequently regulated by environmental cue and through complex hormonal networks. Animals can receive environmental cues and regulate diapause entering and maintenance via a complex hormone network. The source of hormones might differ between mammals (from the mothers) and non-mammals. Note that the same hormone network might be utilized differently or even in the opposite way (i.e. the mouse and mink here). Hormones not involved in regulation are grayed out. JHs—juvenile hormones; 20HE—20-hydroxyecdysone; PTTH—prothoracicotropic hormone. References: mouse [99,171], mink [125–127], mosquito [45,153,154].

As a response to environmental pressure, diapause programmes can be regulated by signals from the environment and act accordingly for its entering and exiting decisions to align animals’ active lives with favourable conditions. Historically, early studies mostly focused on finding systemic factors that may regulate diapause. Hormones emerged as one of the earliest factors identified to mediate the communications from environment to animals [5]. In both invertebrates and vertebrates, changes in hormone levels during development directly impact the ability to enter and exit diapause [152]. While different species may rely on distinct signals and cues to regulate diapause, there are also similarities in features conserved across species (figure 2).

Among invertebrates, the interplay between hormones and diapause is well understood in insects. The transition into diapause in many insect species is regulated by the crosstalk between three key hormones: juvenile hormones (JHs), 20-hydroxyecdysone (20-HE) and prothoracicotropic hormone (PTTH) [172]. During normal development, the presence of JHs (downstream of the insulin pathway) prevents reproductive diapause from occurring [50,173,174]. While the full pathway remains unclear, increased JHs prevent diapause by inhibiting FOXO, which is required for diapause [156]. The second regulatory hormone, 20-HE, plays an antagonistic role towards JHs [175]. 20-HE promotes metamorphosis. Its decreasing levels during pupal and larval stages often trigger diapause [176–178]. Generally, 20-HE levels are directly correlated with the photoperiod. Longer days keep 20-HE at a higher level (promoting metamorphosis). Shorter days, on the other hand, lead to lower 20-HE synthesis (preventing metamorphosis) and enhance the initiation of diapause [132,179]. The last diapause regulatory hormone, PTTH, acts as a mediator between JH and the ecdysone receptor [133]. JH inhibits the neurosecretory cells releasing PTTH, lowering the sensitivity to ecdysone during diapause [134]. While PTTH does not seem to have a clear role in initiating diapause, it does seem essential in maintaining the diapause state in insects, as the PTTH loss-of-function mutants are unable to stay in diapause long-term [135].

A similar interplay of hormones to regulate the entry and exit of diapause also exists in mammals. Since mammalian diapause occurs within the mother, these hormones are usually maternally deposited to the embryos rather than originating from the embryos themselves (figure 2). The interplay between three hormones—prolactin, progesterone and estrogen—regulates the entry and exit of diapause across both lactational (facultative) and seasonal (obligate) forms of diapause [99,180]. However, very interestingly, the same set of hormones are capable of operating in opposite directions depending on the biological context and the species (figure 2, lactational versus seasonal diapause in mouse and mink). During lactational diapause, when the mother has suckling newborns, prolactin levels typically increase [171]. This leads to a decrease in the amount of progesterone and estrogen circulating through the mother [99]. In mice, this interplay of hormones prevents embryo implantation into the uterus and subsequently leads to diapause [28,181]. This process can be completely terminated by a single injection of estrogen into the mother [121]. Conversely, during the seasonal diapause of many mammals, such as the mink (*Mustela vison*), decreasing prolactin is essential for diapause entry [125,128,182]. However, this change in prolactin is linked to photoperiod—not lactation [29,183]. During winter, as the amount of daylight decreases, melatonin increases. This in turn lowers the prolactin levels, leading to diapause entry [183]. Conversely, when the days start to lengthen, melatonin levels start to decrease, leading to elevated levels of prolactin. This increase in prolactin subsequently breaks embryos out of diapause and allows them to be implanted into the uterus and resume embryogenesis [29,126]. This process can be prevented by artificially giving the mother chronic shots of melatonin [127].

Remarkably, there may be some conserved hormonal mechanisms across both invertebrates and vertebrates for diapause regulation. In *C. elegans*, key signalling pathways such as the insulin/IGF-1, TGFb and cGMP signalling pathways converge on the nuclear receptor (DAF-12) to determine whether *C. elegans* enters the dauer stage [184]. Similarly, *D. melanogaster* relies on ecdysone receptor signalling to integrate environmental signals to regulate adult reproductive diapause [185]. Recently, similar mechanisms have been found in vertebrates. The vitamin D receptor, the vertebrate homologue of DAF-12, can also regulate the developmental trajectory of the embryos of South American ocellaris killifish *A. limnaeus*. Treating killifish embryos with exogenous vitamin D prevents them from entering diapause [129]. Unlike mammals, fish embryos are developed independently outside the female body. The natural source of vitamin D in preventing killifish diapause remains unclear, as to whether it is synthesized by the embryos or comes from the environment.

## 8. Diapause preserves resources and energy by managing key nutrient pathways of metabolism

**Figure 3.**
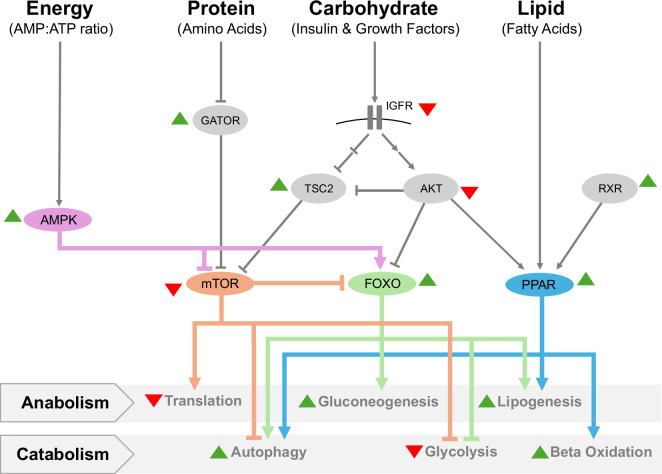
Diapause preserves energy and resources through key metabolic regulators AMPK, mTOR, FOXO and PPAR. Animals preserve resources during diapause by shifting from anabolic to catabolic pathways during diapause. Many aspects of these pathways are conserved across various species. All selected proteins are documented to function during diapause in at least one species, while not necessarily all in the same species. Green and red arrows indicate upregulation and downregulation, respectively. References: [39,119,122,123,136,186–189].

The fundamental function of diapause is to conserve resources and energy during unfavourable conditions, with the anticipation of more favourable conditions ahead. Unsurprisingly, a significant portion of identified molecular pathways associated with diapause are involved in nutrient and metabolic management. This metabolic adaptation of diapause frequently features a global downregulation of metabolic rate, a shifted preference for nutrient-scavenging pathways and a changed oxidative priority of nutrients (figure 3).

Metabolic depression is one of the hallmarks of diapause. Diapause is typically characterized by a significant reduction in metabolic rates between 1% and 40% compared to the non-diapause state [130,190]. Despite this dramatic reduction in metabolism during diapause, embryos maintain essential biological activity sufficient to sustain life for several years, as exemplified by the killifish [106,191,192]. This is achieved by utilizing nutrient sensing and managing pathways to finetune and match their growth and metabolic rates with available resources [193]. Key pathways, including insulin/IGF (sensing growth hormones/carbohydrates), mTOR (sensing amino acids) and PPAR (sensing lipids), regulate downstream effectors to modulate metabolism [193] (figure 3). During diapause, the insulin/IGF and mTOR pathways are typically suppressed while the PPAR pathway is activated to maintain essential biological activities [119,122,131,138]. Suppressing the insulin/IGF pathway triggers a signalling cascade that eventually results in FOXO nuclear translocation leading to the upregulation of various stress-resistance and energy management genes which lower the overall metabolic rate [38,82]. In parallel, suppressing mTOR signalling leads to decreased cellular growth and protein synthesis also resulting in a decreased metabolic rate [122,186]. While suppressing the insulin/IGF and mTOR pathways downregulates the overall metabolic rate, a baseline level still needs to be kept to maintain essential biological activities. One way is through the PPAR pathway, which is involved in lipid metabolism [194]. Upregulating PPARα stimulates the utilization of fatty acid oxidation during diapause by facilitating the breakdown of lipids [194,195]. Collectively, these pathways lead to metabolic depression by shifting from anabolic processes (e.g. biosynthesis) to catabolic processes (e.g. biodegradation) during diapause (figure 3).

Resource recycling is heavily favoured during diapause. This enables organisms to survive prolonged periods of dormancy by efficiently managing their limited energy and nutrient reserves [196]. As such, there is a shift from the energy-intensive ubiquitin-proteosome system (proteasome) towards autophagy for bulk degradation and recycling during diapause [196]. This shift conserves energy while still maintaining essential biological activity for survival, particularly regarding protein metabolism. Autophagy typically occurs via several mechanisms: macroautophagy (the major form), microautophagy and chaperone-mediated autophagy [197,198]. Macroautophagy engulfs cellular components and delivers them to lysosomes for bulk degradation [199]. This process is tightly regulated by AMPK, which modulates the activity of key autophagy-related proteins during diapause [199]. For example, during the embryonic diapause of brine shrimp (*Artemia parthenogenetica*), autophagy-promoting genes *atg5* and *atg8* are upregulated [39]. Furthermore, in the killifish, autophagy-related genes are significantly upregulated during diapause [106]. Beyond protein metabolism, autophagy affects other cellular processes as well. Mitophagy (degrading mitochondria) becomes more prominent during diapause [200]. Mitophagy breaks down and recycles damaged or dysfunctional mitochondria, preventing the build-up of harmful reactive oxygen species (ROS) and maintaining cellular health [201]. For example, in the beetle *L. decemlineata*, the mitophagy-related genes *parkin* and *atg5* are significantly upregulated [200]. Nucleic acid recycling is also required for maintaining cellular functions during diapause. Ribophagy (degrading ribosomes) helps recycle nucleic acids into metabolites needed for survival [202]. While there is some evidence of increased ribophagy during diapause, particularly in *Atemia*, the mechanisms of this process remain elusive [39]. The overall shift from the proteosome to autophagy is coordinated by a complex network involving nutrient-sensing pathways such as AMPK and mTOR [203]. During low-energy conditions, AMPK inhibits mTORC1 promoting autophagy [122,203]. This switch prioritizes resource recycling over biosynthesis during diapause.

Diapause also utilizes a scheme distinct from the active state in managing the intracellular energy. It prompts a metabolic shift away from carbohydrate and protein dependence towards lipids as the primary fuel source [38,123]. During normal development, carbohydrates and proteins are typically prioritized for oxidation while lipids are utilized for long-term storage [193]. In animals, proteins cannot be actively stored as a long-term energy source. While carbohydrates can be stored as glycogen in the liver and muscle, there is a limit to the amount that can be stored [204]. Lipids, meanwhile, do not have that limitation and are approximately twice as calorie-rich [205]. Significant metabolic depression and increased resource recycling during diapause mean that the slow-burning highly dense lipid is favoured. In fact, lipid droplets are essential for the survival of mammalian embryos during diapause [51,124,206]. The intracellular lipid droplets also appear to have nutrient roles in the diapause states beyond mammals, including many insects (such as *D. melanogaster* and *A. albopictus*) and killifish (such as *A. limnaeus* and *N. furzeri*) [51,77,119,157,207]. Lipidomic studies of the African turquoise killifish (*N. furzeri*) embryos further suggested that very-long-chain triglycerides (energy-dense lipids) are being used as the energy source during diapause, coupled with an increased expression of genes involved in their metabolism (e.g. *lpin1*) [119]. In both invertebrates and vertebrates, this switch to fatty acid metabolism is particularly mediated by FOXO transcription factors [119,155,208,209], with the downstream target genes involved in both upregulating lipid metabolism and suppressing other metabolic pathways (noticeably the TCA cycle and pyruvate metabolism) [155,208]. Importantly, while this fatty acid-preferred metabolism is commonly adopted in diapause, different animals might implement this metabolic modulation differently, depending on the nature of their development and the timing of their diapause. Some species incorporate nutrients from external or maternal sources during development and thus can directly link this modulation to the nutrient level from the environment. For example, *C. elegans* intakes external nutrients during development. When there is a decrease in nutrient availability during development, the insulin/IGF pathway responds accordingly and is suppressed [38,193]. DAF-2 (insulin/IGF receptor) becomes inactive and through its immediate downstream protein AGE-1 (PI3K) and a signalling cascade, DAF-16 (FOXO) enters the nucleus and turns on the expression of target genes promoting dauer formation [138,149,210]. In mice, hormones released during lactational diapause ultimately lead to fewer macronutrients within the uterine fluid which activates TSC-2 and GATOR1, inhibiting mTORC1 (mTOR complex 1, the key protein complex that mTOR forms to regulate downstream targets) [123]. Furthermore, artificially inhibiting mTOR in mouse blastocysts and mESCs can induce diapause [186], in which FOXO1 was shown to be essential for mediating the transition to fatty acid metabolisms [122,186,208]. On the other hand, there are also animals developing with fixed amounts of nutrients deposited in the eggs and thus the metabolic modulation during embryonic diapause is not directly linked to nutrient scarcity. In these cases, such as killifish and insect eggs, the modulation occurs downstream of nutrient sensing, for example, downregulating the expression of insulin/IGF receptor and the pathway directly in diapause [131,211]. Furthermore, in killifish (*N. furzeri*) and mosquitoes (*C. pipiens*), FOXOs have also been indicated to be essential for mediating the metabolic transition to fatty acid oxidation [45,119].

## 9. Diapause can help reveal the general principle of biological dormancy

Diapause is a fascinating phenomenon and one of many forms of biological dormancy. Dormancy, manifesting in various forms from quiescence in stem cells to hibernation for winter animals to dormant cancer cells after chemotherapy, shares the same purpose of preserving life during harsh periods. It encompasses shared features such as arrested cell proliferation, lower metabolic rates, improved protein homeostasis, enhanced stress resistance and efficient resource and energy management [212–216]. It is thus important and of great interest to know if there exists a universal molecular core programme for various dormant states to build on.

Our increasing understanding of different diapause states at the molecular level provides a valuable opportunity to identify the genetic basis of the shared and unique features between different types of dormancy. For example, drug-resistant colorectal cancer cells show striking similarities to the gene signature of blastocysts undergoing diapause, including epigenetic changes [166,216]. These parallels suggest that the same pathways regulating diapause could be involved in these diapause-like cancer cells, allowing them to survive treatment and potentially contribute to relapse. Similarly, quiescent stem cells rely on FOXO proteins to maintain their cellular integrity and pluripotency [217–219]. FOXO transcription factors are essential for regulating cell-cycle arrest and protecting cells from oxidative stress. For example, mice lacking FOXO3 cannot maintain their neural stem cells into adulthood [220]. Furthermore, there is a metabolic shift towards fatty acid oxidation and autophagy rather than glycolysis and proteosomes during quiescence to maintain energy levels, like many diapause states [213]. This metabolic adaptation is observed in both cancer cells and stem cells, highlighting the conservation of this survival mechanism across different cell types and dormancy. The crosstalk between these pathways contributes to the metabolic reprogramming that allows cells to survive in a diapause-like state, indicating the translational value of potential new targets for therapeutic interventions in cancer treatment and stem cell regeneration [203].

The conservation between dormancy highlights the importance of the key genetic components comprising the core functions of biological dormancy. Diapause should thus serve as a good model for understanding and potentially targeting other forms of dormancy.

## Data Availability

This article has no additional data.
